# Curcumin-Functionalized Ag and ZnO Nanoparticles: A Nanotherapeutic Approach for Treating Infections in Diabetic Wounds

**DOI:** 10.3390/bioengineering12101090

**Published:** 2025-10-09

**Authors:** Mahboubeh Dolatyari, Parisa Rostami, Mahya Rostami, Ali Rostami, Hamit Mirtagioglu

**Affiliations:** 1 SP-EPT Lab., ASEPE Company, Industrial Park of Advanced Technologies, Tabriz 5364191686, Iran; 2 Student Research Committee, Tabriz University of Medical Science, Tabriz 5166614766, Iran; 3 Research Center for Evidence-Based Medicine, Iranian EBM Centre, A JBI Centre of Excellence, Faculty of Medicine, Tabriz University of Medical Sciences, Tabriz 5166614766, Iran; 4 Faculty of Medicine, Dokuz Eylul University, Izmir 35660, Turkey; 5 Photonics and Nanocrystal Research Lab. (PNRL), University of Tabriz, Tabriz 5166614761, Iran; 6 Department of Statistics, Faculty of Science and Literature, University of Bitlis Eren, Bitlis 13100, Turkey

**Keywords:** wound healing, nanoparticles, molecular docking, curcumin, dressing pad, ointment

## Abstract

Chronic wounds, such as diabetic ulcers, remain a significant clinical challenge due to high infection risk and delayed healing. This study presents a comprehensive evaluation of a novel wound dressing incorporating curcumin-functionalized silver–zinc oxide (Ag-ZnO) nanoparticles. The formulation was rationally designed based on molecular docking simulations that identified curcumin as a high-affinity ligand for *Staphylococcus aureus* Protein A. The synthesized nanoparticles demonstrated potent, broad-spectrum antibacterial activity, achieving complete inhibition of multidrug-resistant pathogens, including MRSA, within 60 s. A critical comparative assessment, incorporating an unloaded Ag-ZnO nanoparticle control group, was conducted in both a rabbit wound model and a randomized clinical trial (*n* = 75 patients). This design confirmed that the enhanced wound-healing efficacy is specifically attributable to the synergistic effect of curcumin combined with the nanoparticles. The curcumin-loaded Ag-ZnO treatment group showed a statistically significant reduction in healing time compared to both standard care and unloaded nanoparticle controls (e.g., medium wounds: 19.6 days vs. 90.6, *p* < 0.001). These findings demonstrate that curcumin-functionalized Ag-ZnO nanoparticles offer a safe and highly effective therapeutic strategy, providing robust antibacterial action and significantly accelerated wound healing.

## 1. Introduction

Chronic wounds represent a significant global healthcare challenge due to their prolonged healing process, often complicated by infections and poor wound closure [1,2,3]. These wounds have an increasingly significant economic impact, particularly in developed countries, as their prevalence rises in tandem with an aging population. Current treatment options often fail to fully resolve these injuries, highlighting the need for innovative therapies that promote healing, prevent infections, and reduce inflammation [4,5]. Nanotechnology-based approaches have emerged as promising alternatives, offering multifunctional solutions to enhance wound healing, as demonstrated in various animal models. These advances are paving the way for next-generation wound nanotherapies [1,2,3,4,5]. Among chronic wounds, non-healing pressure ulcers (NHPUs), venous ulcers (VUs), and diabetic foot ulcers (DFUs) are the most prevalent [6,7,8,9,10]. DFUs, in particular, are associated with diabetes-related complications such as neuropathy, peripheral arterial disease, and foot deformities. These complications contribute to poor wound closure, heightened risk of infection, and, in severe cases, amputation. Chronic wounds not only impose a physical and emotional burden on patients but also significantly impact their families and healthcare systems [11,12,13,14,15,16,17,18,19,20,21,22]. Medicinal plants have long been a cornerstone of traditional medicine, offering a rich repository of bioactive compounds with therapeutic potential [23,24,25,26]. Recent advances in computational drug discovery have further accelerated the identification of such compounds. Techniques such as virtual screening, molecular docking, and density functional theory (DFT) analysis allow researchers to efficiently explore the interactions between bioactive molecules and their target proteins. These in silico methods complement traditional experimental approaches, enabling the rapid discovery of novel therapeutic agents [27]. Curcumin, a natural polyphenol derived from *Curcuma longa*, has been extensively studied for its wound-healing properties. It has demonstrated antibacterial, antioxidant, and anti-inflammatory activities while promoting key processes, such as fibroblast migration, collagen synthesis, and tissue remodeling [28,29,30,31,32]. Recent studies underscore its efficacy in modulating inflammatory responses and enhancing tissue proliferation, making it a potent agent for managing chronic wounds [33]. Advancements in wound management have also seen the integration of synthetic dressings and nanoparticle (NP)-based therapies. Metallic nanoparticles, including those composed of silver and zinc oxide, exhibit potent antimicrobial properties and promote tissue repair. These nanoparticles function by generating reactive oxygen species (ROS) and disrupting bacterial cell components, making them particularly effective against resistant pathogens [31,32,33]. However, optimizing their formulations to maximize efficacy while minimizing toxicity remains a key research focus [34,35,36,37,38,39,40,41,42,43]. Recent studies have investigated the combination of silver nanoparticles with curcumin for wound healing. For example, curcumin–silver nanofibers accelerate closure and reduce bacterial load [44], while guar gum/curcumin–silver hydrogels improve tissue regeneration [45]. Green-synthesized curcumin–silver nanoparticles in bacterial cellulose have also shown promise for antimicrobial wound dressings [46]. Our work investigates a hybrid system of Ag-ZnO nanoparticles functionalized with curcumin. This combination leverages the synergistic antibacterial effect between silver and zinc oxide, allowing for a reduction in the required concentration of silver nanoparticles and potentially enhancing biocompatibility.

In this study, we explore the wound-healing potential of curcumin-encapsulated silver and zinc oxide nanoparticles. Computational docking studies were employed to investigate the interactions between *Staphylococcus aureus* cell wall proteins and curcumin, as well as 1-(4-methylphenyl)-3-(3-nitrophenyl)-2-propen-1-one, a key compound found in aloe vera gel. Based on promising simulation results, curcumin-modified Ag-ZnO nanoparticles were synthesized and utilized in the preparation of an ointment and dressing pad. These products were subsequently tested for their therapeutic efficacy in wound healing, offering a novel approach to managing infections and promoting tissue repair in diabetic foot ulcers.

## 2. Materials and Methods

### 2.1. Chemical

The used reagents and chemicals are listed in Table 1.

### 2.2. Molecular Operating Environment (MOE) Docking Methodology

The active site of the receptor protein was identified using MOE software Version 2019.01 (Chemical Computing Group ULC, Montreal, QC, Canada). A molecular docking approach was employed with MOE to screen a library of 1000 phytochemicals against the protein’s interacting residues. The software confirmed the optimal ligand conformation for constructing a minimum-energy structure using the “Triangular Matcher” algorithm, which served as the default ligand insertion strategy [47]. Simulated poses were rescored using the London dG scoring function within MOE. Following docking, the top-ranking phytochemicals were selected based on RMSD values and binding affinity (S-score). The MOE LigX tool facilitated visualization of the best-docked complexes and generated 2D interaction plots of ligand–receptor binding. Additionally, MOE provided three-dimensional visualizations of protein–ligand complexes.

### 2.3. Synthesis of Ag-ZnO Nanoparticles

The synthesis of Ag-ZnO involved dissolving 0.072 g of silver acetate and 0.216 g of polyvinylpyrrolidone (PVP) in 450 mL of deionized water. The solution was stirred for 5 min. Then, 0.03 g of sodium borohydride was dissolved in 50 mL of deionized water and added dropwise to the first solution. The mixture was stirred for 30 min. Next, 0.33 g of zinc acetate dihydrate was introduced into the solution, followed by the gradual addition of another 0.1 g of sodium borohydride dissolved in 500 mL of deionized water. This procedure resulted in the formation of nanoparticles containing 45 ppm of silver (Ag) and 150 ppm of zinc oxide (ZnO) [48]. The reaction mixtures were stirred using a magnetic stirrer (IKA, Staufen, Germany). The stirring speed was maintained at 600 rpm, which ensured homogeneous mixing of all reagents during nanoparticle synthesis.

### 2.4. Interaction of Synthesized Nanomaterials with Curcumin

The interaction between the synthesized nanomaterials and curcumin was achieved by dissolving 0.5 g of extracted curcumin in 20 mL of absolute ethanol, followed by the addition of 0.2 g of NaOH dissolved in 20 mL of water. The resulting solution was then combined with 80 mL of the synthesized colloidal nanoparticles. The mixture was stirred continuously for 24 h, yielding the materials utilized as a wound-healing matrix.

### 2.5. Preparation of Ointment

To prepare the ointment, a mixture of 10 g of 1-hexadecanol and 20 g of solid linseed oil was melted using a water bath. Simultaneously, 0.5 g of synthesized nanomaterials was dispersed in 70 g of water and heated to 70 °C. Afterward, the two liquids were blended and stirred vigorously until they cooled down to room temperature. The final product had a texture similar to that of a cream.

### 2.6. Preparation of Wound Pad

To prepare the wound pad, a 1% PVA solution was prepared by adding 0.5 g of carboxymethyl cellulose (CMC) and 0.5 g of synthesized nanomaterials to 100 mL of the solution. The mixture was thoroughly stirred until it formed a thick, viscous solution. Next, the solution was dried, and the resulting film was carefully cut into 10 × 10 pieces and packed with utmost care.

### 2.7. Instrumentation

Transmission electron microscopy (TEM) was performed using a Philips EM 208S instrument (Philips, Amsterdam, The Netherlands) to evaluate the morphology and size distribution of the synthesized nanoparticles. The hydrodynamic particle size and polydispersity index (PDI) of the nanoparticle formulations were determined by dynamic light scattering (DLS) using a VASCO DLS system (Scimed, Stockport, UK). Fourier-transform infrared (FT-IR) spectra were recorded with an MB3000 spectrometer (ABB, Zürich, Switzerland) to confirm surface functionalization and ligand interactions. Photoluminescence spectra were obtained using an LS 55 Luminescence Spectrometer (PerkinElmer Inc., Rodgau, Germany) to investigate the optical properties of the nanoparticles.

### 2.8. In Vivo Safety and Efficacy Evaluation in a Rabbit Model

− *Irritation, Systemic Toxicity, and Wound-Healing Assessment:*

The biocompatibility and wound-healing efficacy of the formulations were evaluated in vivo using healthy adult white albino rabbits. The study was conducted at the Cell and Animal Toxicology Laboratory, Faculty of Pharmaceutical Sciences, University of Tehran, in accordance with international guidelines (ISO 10993-10 for skin irritation [49] and ISO 10993-11 for systemic toxicity [50]). The protocol was approved by the Research Ethics Committee of Tabriz University of Medical Sciences (Approval ID: IR.TBZMED.REC.1399.1169). To comprehensively address the role of each component, a full-thickness excisional wound model was employed. Wound depth (~1 mm) and the wound area (~2 cm^2^) were generated during the irritation test on rabbits. Each wound received a different treatment, allowing for direct intra-animal comparison:

Group 1 (Untreated Control): Wounds cleansed with saline and covered with sterile, non-medicated gauze.

Group 2 (Unloaded Ag-ZnO-curcumin Nanoparticles): Wounds treated with an ointment containing unloaded Ag-ZnO nanoparticles.

Group 3 (Treatment): Wounds treated with the Ag-ZnO-curcumin nanoparticle ointment.

We covered all wounds with sterile dressings. Patients were monitored at regular intervals until the wound had closed. For the irritation test, the formulations were applied to shaved skin under semi-occlusive conditions and observed at 24, 48, and 72 h for erythema and edema, scored using a standardized scale. For systemic toxicity assessment, animals were monitored for 14 days for clinical signs, body weight, and food intake.

The animal experiments in our study were designed primarily to evaluate the biocompatibility, irritation potential, and preliminary wound-healing capacity of the synthesized Ag-ZnO-curcumin nanoparticles under controlled physiological conditions. While the irritation-induced wounds in rabbits do not fully replicate diabetic wound pathology, this model was intentionally selected as a first-step in vivo safety and efficacy assessment, in line with ISO 10993-10 and ISO 10993-11 standards.

### 2.9. Clinical Trial Design

A randomized clinical trial was conducted to evaluate the wound-healing efficacy of Ag-ZnO-curcumin nanoparticle-based dressings in a human population. A total of 75 participants with comparable chronic ulcers were enrolled and randomly divided into three groups (*n* = 25 per group):

Group A (Standard Care Control): Wounds cleansed with baby shampoo and betadine, then dressed with a sterile dressing.

Group B (Unloaded Ag-ZnO-Curcumin Nanoparticles): Wounds received the same cleansing protocol, followed by the application of pads and ointments containing unloaded Ag-ZnO-curcumin nanoparticles. These wounds were dressed with a Vaseline-coated PVA-carboxymethyl cellulose pad.

Group C (Treatment Group): Wounds received the same cleansing protocol followed by application of pads and ointments containing Ag-ZnO-curcumin nanoparticles.

The trial was approved by the Research Ethics Committee of Tabriz University of Medical Sciences (IR.TBZMED.REC.1399.1169) and registered in the Iranian Registry of Clinical Trials (IRCT20190701044062N9). All participants provided written informed consent. Exclusion criteria were as previously detailed.

### 2.10. Bacterial Growth Inhibition Test

Five bacterial strains were tested in this study: *Escherichia coli*, *Staphylococcus aureus*, *Enterococcus hirae*, *Pseudomonas aeruginosa*, and Methicillin-resistant *Staphylococcus aureus* (MRSA). All bacterial strains were obtained from MikroBank at the University of Tehran, Iran. Bacterial cultures were incubated for 24 h at 37 °C, and colony-forming units per milliliter (CFU/mL) were determined, with an initial bacterial concentration of approximately 1.5 × 10^8^ CFU/mL.

To evaluate the antibacterial effect, 0.3 g/L of bovine serum albumin (as an interfering substance) was added to 1 mL of bacterial suspension and allowed to stand for 2 min at 20 °C. Following this, 8 mL of the synthesized colloidal nanoparticles was introduced into the suspension. A neutralizing agent containing 30 g/L polysorbate, 30 g/L saponin, and 3 g/L lecithin was used to ensure accurate assessment of bacterial viability. As a negative control, an equal volume of bacterial suspension was mixed with deionized water. All samples were then incubated overnight in a shaking incubator under identical conditions to assess bacterial inhibition.

### 2.11. Ethics Approval and Consent to Participate

Hereby, we confirm that all experiments were conducted in accordance with relevant guidelines and regulations. Toxicity and irritation tests were studied at the Cell and Animal Toxicity Laboratory, Faculty of Pharmacy, University of Tehran Medical Sciences, Tehran, P.O.Box: 14155/6451, I.R. IRAN. The standards for irritation and toxicity experiments and methods were ES, BN ISO 10993: 10; 2016, and BS EN ISO 10993-11(2009). All experimental protocols were approved by the IR-FDA and Research Ethics Committees of Tabriz University of Medical Sciences with Approval ID: IR.TBZMED.REC.1399.1169 issued on 15 March 2021. This study is registered at https://irct.behdasht.gov.ir under trial registration number IRCT20190701044062N9, registered on 11 October 2021. We confirm that informed consent was obtained from all subjects or their legal guardians. All methods were carried out according to relevant guidelines and regulations. The study is reported in accordance with the Animal Research: Reporting of In Vivo Experiments (ARRIVE) guidelines. All experimental procedures involving animals were conducted according to institutional, national, and international guidelines, including the Basel Declaration and the ethical principles for animal research. Anti-bacterial test methods were performed in accordance with EN 1276. All participants in this study were provided with a detailed informed consent form before enrollment. The form explained the study objectives, procedures, potential risks, and benefits. Written informed consent was obtained from all participants, and their voluntary agreement to participate was documented with signatures. The study protocol, including the consent process, was reviewed and approved by the Ethics Committee of Tabriz University of Medical Sciences with Approval ID: IR.TBZMED.REC.1399.1169 issued on 15 March 2021.

## 3. Results and Discussion

**Figure 1:**
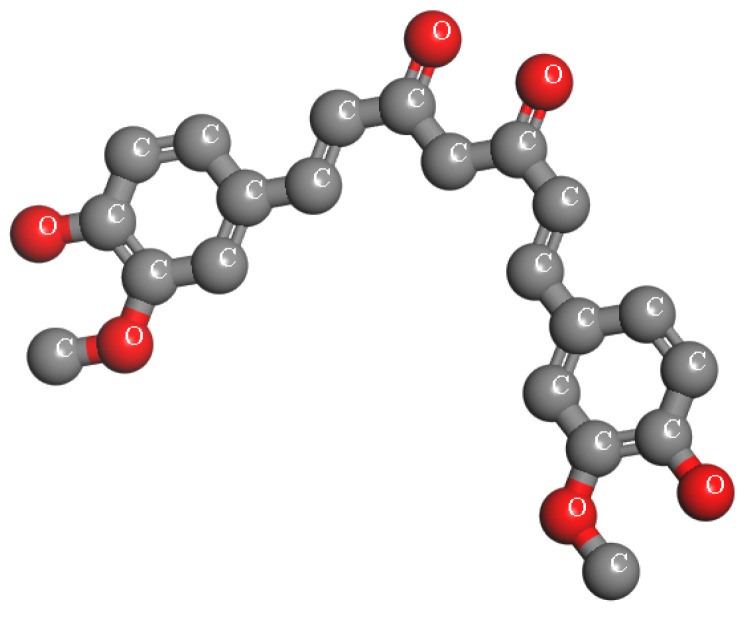
The chemical structure of Curcumin.

Curcumin, a symmetric molecule also known as diferuloylmethane, has the IUPAC name (1E,6E)-1,7-bis(4-hydroxy-3-methoxyphenyl)-1,6-heptadiene-3,5-dione, a molecular formula of C_21_H_20_O_6_, and a molecular weight of 368.38. Its structure includes two aromatic rings with o-methoxy phenolic groups connected by a seven-carbon α,β-unsaturated β-diketone linker (see Figure 1). Curcumin undergoes keto-enol tautomerism, with the enol form being more stable in most solvents due to resonance-assisted hydrogen bonding and extended conjugation. In crystal form, curcumin adopts a cis-enol configuration, while in solution, it exists as cis-trans isomers, with the trans form being slightly more stable. The molecule is hydrophobic, with a logP value of ~3.0, making it nearly insoluble in water but readily soluble in polar solvents such as DMSO, ethanol, and methanol. Its dipole moment in the ground state is 10.77 D, and it is sparingly soluble in hydrocarbons like cyclohexane and hexane [51].

**Figure 2:**
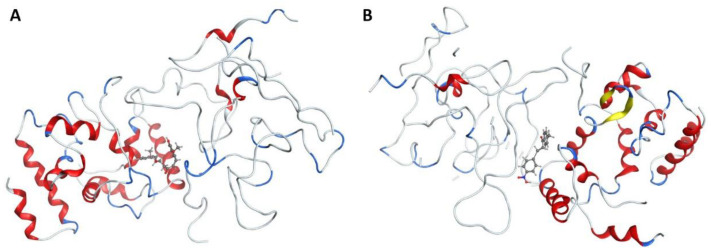
Interaction between cell wall protein of *Staphylococcus aureus* and (**A**) Curcumin and (**B**) 1-(4-methyl phenyl)-3-(3-nitrophenyl)-2-propane-1-one. α-helices (red ribbons): Cylindrical coiled structures. ClfA has several helices forming the overall IgG-like fold of the N2 and N3 domains. β-sheets (yellow ribbons): These form the immunoglobulin-like β-sandwich fold, essential for ligand recognition. Loops/turns (gray ribbons): Connect helices and sheets; some are flexible and contribute to binding. Ligand peptide (blue ribbons): A short fibrinogen γ-chain segment bound between the N2 and N3 domains. N2 domain: Immunoglobulin-like domain, part of the “dock, lock, latch” (DLL) mechanism of fibrinogen binding. N3 domain: Complements N2 in ligand binding; together they clamp the fibrinogen γ-chain peptide.

In this study, molecular docking was employed to evaluate the potential of curcumin and 1-(4-methylphenyl)-3-(3-nitrophenyl)-2-propen-1-one, a key component of Aloe vera gel, as effective agents for surface modification of Ag and ZnO nanoparticles for healing diabetic foot wounds. The interactions of these compounds and their nanoparticle-bound forms with the cell wall protein of Staphylococcus aureus were investigated (see Figure 2). This bacterium is a primary pathogen responsible for diabetic wound infections.

**Figure 3:**
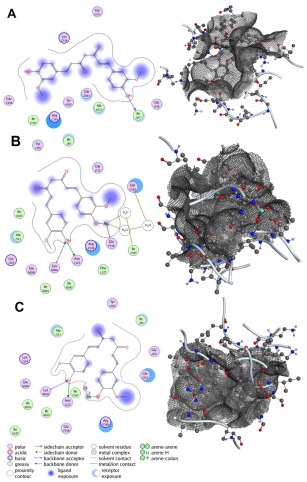
Interaction patterns and surface mapping of Protein A (cell wall protein of *Staphylococcus aureus*) with (**A**) Curcumin, (**B**) surface-modified Ag, and (**C**) ZnO functionalized with Curcumin. The left panel represents the 2D interaction diagram between the ligand and amino acid residues of Protein A. The right panel shows the corresponding 3D surface representation of the binding pocket, where the ligand is visualized within the protein’s active/binding site. This dual presentation illustrates both the molecular interactions (left) and the spatial orientation within the binding cavity (right).

**Figure 4:**
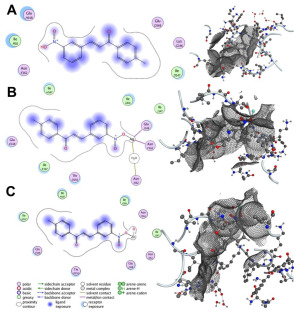
Interaction patterns and surface mapping of Protein A (cell wall protein of Staphylococcus aureus) with (**A**) 1-(4-methylphenyl)-3-(3-nitrophenyl)-2-propane-1-one, (**B**) surface-modified Ag, and (**C**) ZnO functionalized with 1-(4-methylphenyl)-3-(3-nitrophenyl)-2-propen-1-one. In each subfigure, the left panel displays the 2D interaction diagram, highlighting hydrogen bonds, hydrophobic interactions, and other significant binding forces between the ligand and amino acid residues of Protein A. The right panel presents the 3D surface representation of the protein binding pocket, showing the ligand docked within the protein’s active site.

The primary cell wall protein (protein A) plays a crucial role in the pathogenicity of *Staphylococcus aureus* (*S. aureus*). Proteins of the MSCRAMM (microbial surface components recognizing adhesive matrix molecules) family are critical for bacterial adherence to host tissues, facilitating infection [52]. Specific active binding regions of protein A, including residues such as Glu E148, Thr D150, Glu J148, Isn I162, IIe J147, Asn F162, IIeG60, IIe H147, II E147, Asn N62, and others, were identified as essential in ligand–protein interactions (see Figure 3 and Figure 4). These binding pockets were determined using the site finder tool available in the Molecular Operating Environment (MOE) software (version 2019.01).

Surface engineering of nanoparticles plays a crucial role in tailoring their properties for specific applications. To enhance the efficacy of synthesized nanoparticles in combating infections and accelerating wound healing, we functionalized their surface by reacting the dangling bonds with more effective ligands. Curcumin and bioactive compounds extracted from Aloe vera are well-known for their wound-healing properties. To identify the most suitable ligand, we simulated the interaction of Ag and ZnO functionalized with curcumin and 1-(4-methylphenyl)-3-(3-nitrophenyl)-2-propen-1-one with the cell wall protein (protein A) of Staphylococcus aureus. The docking simulations revealed favorable binding scores and RMSD values for the interactions between the selected compounds and protein A. Among the tested compounds, curcumin-bound Ag demonstrated the best binding score, with a computed RMSD value of 0.845, indicating strong alignment and effective interaction. RMSD (Root Mean Square Deviation) is a key metric in molecular docking, used to assess the deviation between the predicted binding pose of a ligand and its experimentally determined native pose. An RMSD value below 2 reflects an intense match between the ligand and the protein’s active site, signifying a reliable interaction [53]. Additionally, Curcumin-, Zinc Oxide-, and Ag-modified compounds exhibited two hydrogen bond interactions with Lys F124 and Glu H148, resulting in stronger bonds and confirming the RMSD values. Among all studied compounds, the binding affinity of Ag modified by Curcumin is −8.424 kcal/mol. It is −4.914, −4.871, 2.945, −5.203, and −3.749 kcal/mol for Curcumin, Curcumin-ZnO, 1-(4-methyl phenyl)-3-(3-nitrophenyl)-2-propane-1-one, 1-(4-methyl phenyl)-3-(3-nitrophenyl)-2-propane-1-one-Ag, and 1-(4-methyl phenyl)-3-(3-nitrophenyl)-2-propane-1-one-ZnO, respectively.

These findings, summarized in Table 2, highlight the high docking performance of curcumin-modified Ag, positioning it as a promising candidate for nanoparticle-based wound therapies. Figure 3 illustrates the key binding interactions, highlighting the active residues involved in this process.

**Figure 5:**
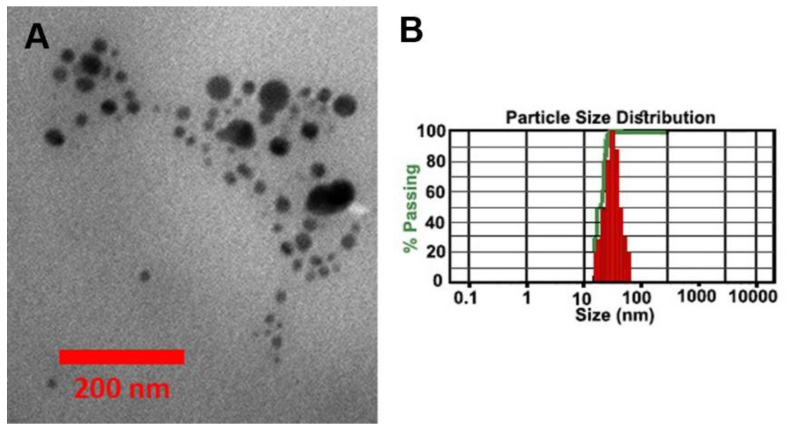
(**A**) TEM image and (**B**) DLS analyses of the synthesized Ag-ZnO nanoparticles modified by Curcumin.

Here, we synthesized Ag and ZnO nanoparticles modified with Curcumin. The TEM image of the synthesized nanoparticles is shown in Figure 5A. The figure shows the spherical nanoparticles with a size distributed between 10 nm and 70 nm. The size distribution of nanoparticles is also measured by DLS Analysis. The result indicates that most particles are approximately 50 nm in size (Figure 5B).

**Figure 6:**
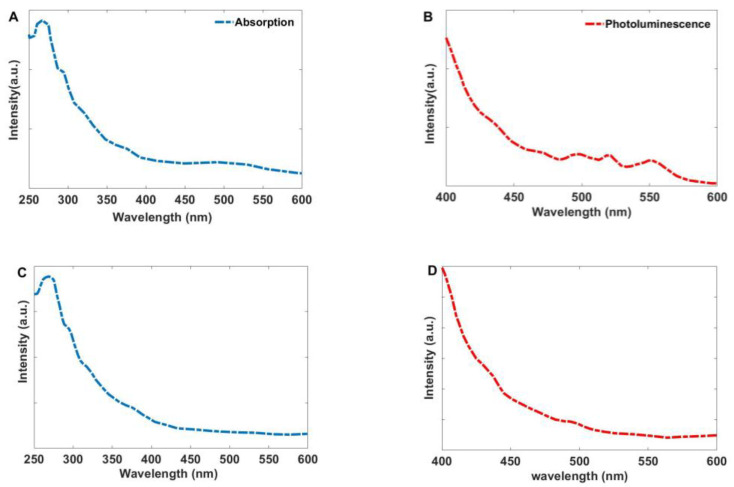
(**A**) The absorption and (**B**) Photoluminescence spectra of the synthesized Ag-ZnO nanoparticles modified by Curcumin, (**C**) Absorption and (**D**) Photoluminescence spectra of the unmodified Ag-ZnO nanoparticles.

The absorption spectrum of the synthesized Ag-ZnO modified by Curcumin is depicted in Figure 6. The figure reveals the presence of absorption bands at 266 nm and 295 nm, along with additional shoulders at 321 nm and 376 nm. Furthermore, the photoluminescence spectrum shows emission bands at 495 nm, 518 nm, and 549 nm. These bands can be related to the coordination of hydroxyl groups from Curcumin ligands on the surface of ZnO and Ag NPs. As shown in Figure 6C,D, the characteristic peaks associated with Curcumin are absent in the spectrum of the Ag-ZnO nanoparticles without Curcumin.

**Figure 7:**
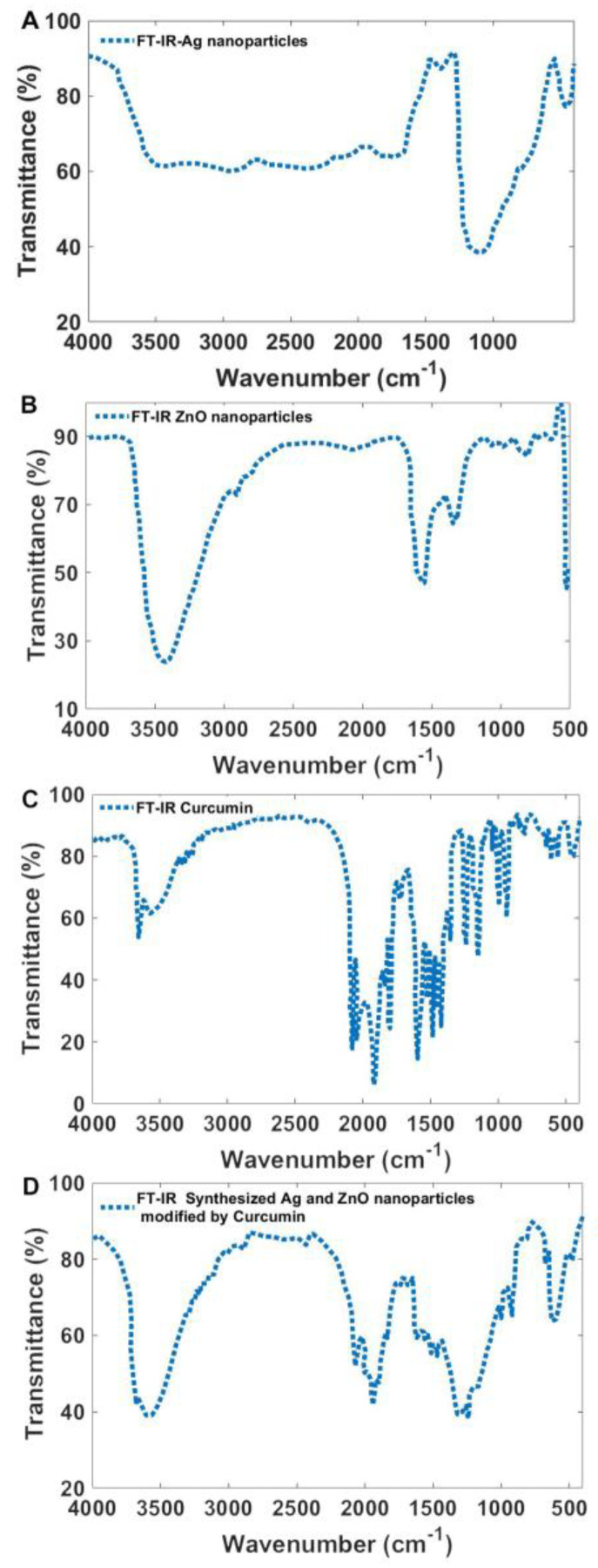
FT-IR spectroscopy of (**A**) synthesized Ag nanoparticles, (**B**) ZnO nanoparticles, (**C**) Curcumin powder, and (**D**) synthesized Ag and ZnO nanoparticles modified by Curcumin.

To assess ligand exchange on the surface of nanoparticles with curcumin, Ag, and ZnO nanoparticles were synthesized separately and analyzed using FT-IR spectroscopy (Figure 7). Additionally, FT-IR analysis was performed on curcumin to characterize its functional groups.

The FT-IR spectrum of Ag nanoparticles displayed characteristic peaks at 3402 cm^−1^, 2930 cm^−1^, 1623 cm^−1^, 1372 cm^−1^, and 1117 cm^−1^. These peaks correspond to O–H stretching (~3402 cm^−1^), C–H stretching (2930 cm^−1^), C=O stretching (1623 cm^−1^), and C–C and O–H stretching (1117 cm^−1^), which are associated with polyvinyl pyrrolidone (PVP) used as a stabilizing agent [54]. For ZnO nanoparticles, absorption peaks were observed at 3438 cm^−1^, 2925 cm^−1^, 1597 cm^−1^, 1388 cm^−1^, 1354 cm^−1^, 1119 cm^−1^, 1006 cm^−1^, 865 cm^−1^, 775 cm^−1^, 700 cm^−1^, and 577 cm^−1^. The peak at 577 cm^−1^ corresponds to metal-oxygen (ZnO) stretching vibrations, while the broadband at 3438 cm^−1^ is attributed to O–H stretching vibrations [55]. The remaining peaks are associated with surface ligands on the ZnO nanoparticles.

The FT-IR spectrum of curcumin exhibited a strong peak at 1628 cm^−1^, attributed to overlapping C=C and C=O stretching vibrations. The spectrum also displayed a broad stretching vibration between 3200–3500 cm^−1^ corresponding to O–H groups, an aromatic C=C stretching peak at 1425 cm^−1^, and a high-intensity band at 1518 cm^−1^, which represents a combination of carbonyl stretching, aliphatic bending vibrations (δ C-C-C, δ C-C=O), and aromatic bending vibrations (δ C-C-H) in both keto and enol configurations. Additionally, a significant peak at 1279 cm^−1^ was assigned to the bending vibration of the phenolic ν(C-O) bond [56,57,58]. As shown in Figure 7D, the characteristic curcumin bands are present in the spectra of the synthesized nanoparticles, confirming the successful substitution of curcumin for most of the original surface ligands. This evidence indicates effective ligand exchange, enhancing the potential bioactivity of the modified nanoparticles.

Numerous studies have provided evidence that silver nanoparticles (Ag-NPs) have a distinct impact on regulating inflammatory processes in wounds and expediting the initial stages of the healing process. This advantageous effect is attributed to the reduction in local matrix metalloproteinase levels and the promotion of apoptosis in cells [59]. Moreover, dressings containing Ag-NPs help to control inflammatory responses, especially when TNF-alpha expression levels are high, leading to anti-inflammatory outcomes [60]. In addition, they stimulate apoptosis, a process recognized for its anti-inflammatory properties, by preventing cells from undergoing necrosis—a characteristic sign of severe inflammation [59].

Table 3 presents the antibacterial effects of the synthesized nanomaterials on the tested bacterial strains. The tested bacterial strains include *Escherichia coli*, *Staphylococcus aureus*, *Enterococcus hirae*, *Pseudomonas aeruginosa*, and Methicillin-resistant *Staphylococcus aureus* (MRSA). The results demonstrate that the growth of all bacteria was entirely inhibited after incubation with the nanoparticles at a concentration of 15 μg/L (Ag)/100 μg/mL (ZnO), modified with 0.5 g/L curcumin. Remarkably, the logarithmic reduction in bacterial count exceeded eight within 60 s, corresponding to nearly 100% inhibition of the bacterial growth. This reduction is well above the acceptable threshold of 5, highlighting the potent antimicrobial activity of the curcumin-functionalized nanoparticles.

**Figure 8:**
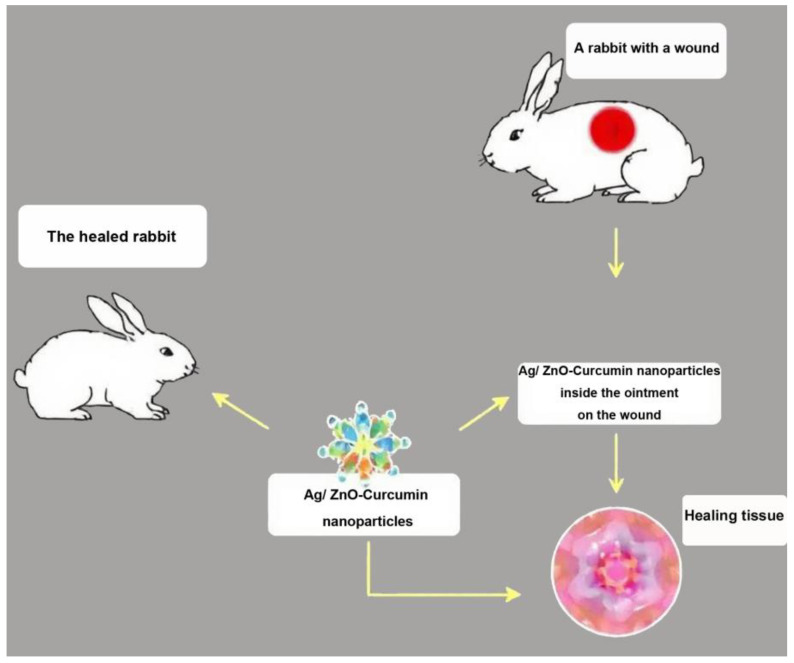
Schematic image showing the effect of nanoparticles on the wound.

**Figure 9:**
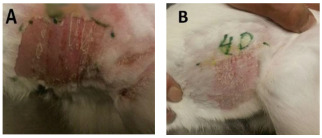
Skin of rabbits (**A**) before, and (**B**) after 4 days using Ag-ZnO nanoparticles modified by Curcumin (ointment and dressing pad).

Figure 8 illustrates a schematic representation of the impact of nanoparticles on wound healing. The prepared ointment and pads were applied to different wounds to assess the effectiveness of treatment using Ag-ZnO nanoparticles modified by Curcumin on the skin of albino rabbits and wounds in humans. No signs of erythema or edema were observed at the application sites over the 72 h observation period for any of the formulations (Untreated control, Unloaded Ag-ZnO, and Ag-ZnO-Curcumin). The Primary Irritation Index (PII) was calculated as 0.00 for all groups, indicating that the formulations were non-irritating. Throughout the 14-day systemic toxicity assessment, all animals exhibited normal behavior, with no significant changes in body weight or food intake compared to their baseline values. Gross pathological examination at the endpoint revealed no abnormalities in major organs, confirming the topical safety of the nanoparticles (see Figure 9).

The wound-healing progress, quantified by the reduction in wound area over time, is summarized in Table 4. The Ag-ZnO-curcumin treatment group (Group 3) exhibited a significantly accelerated healing rate compared to both control groups.

By day 14, wounds treated with Ag-ZnO-curcumin were nearly completely closed (96.8% healing), while the untreated and unloaded nanoparticle groups showed only 69.5% and 79.2% closure, respectively. The time to complete wound closure was significantly shorter (*p* < 0.001) for the Ag-ZnO-curcumin group (12.5 ± 1.3 days) compared to the unloaded nanoparticle group (18.2 ± 1.8 days) and the untreated control (21.4 ± 2.1 days). This result indicates that while the unloaded Ag-ZnO nanoparticles themselves promote healing compared to the untreated control, the incorporation of curcumin provides a synergistic effect, leading to a statistically significant enhancement in healing speed.

The clinical trial results demonstrated a significant and progressive improvement in wound-healing outcomes across the three treatment groups, with the Ag-ZnO-curcumin nanoparticle dressing showing superior efficacy. Analysis of healing rates revealed a clear hierarchy of effectiveness. In the Standard Care Control group (Group A), outcomes were poor, consistent with the challenges of managing chronic wounds. Among the 25 patients, only 15 (60%) achieved any degree of healing within the study period, comprising 10 with soft wounds and 5 with medium wounds. The remaining 10 patients experienced severe complications: 5 with complex wounds showed no healing after 6 to 18 months of follow-up, and 2 of these patients ultimately required leg amputation. A further 5 participants were lost to follow-up. In contrast, Group B, treated with unloaded Ag-ZnO nanoparticles, showed a marked improvement over standard care. This result suggests that the nanoparticle scaffold itself exhibits beneficial wound-healing properties, likely due to its ability to retain moisture and maintain the antibacterial properties of the pad. However, the most pronounced results were observed in the Ag-ZnO-Curcumin Treatment group (Group C). In this group, 22 out of 25 patients (88%) achieved successful and complete wound healing. This result included all patients presenting with soft and medium wounds, as well as 7 out of 10 patients with complex wounds. Only three patients in this group did not complete the study. The graduated efficacy—from Group A (lowest) to Group B (intermediate) to Group C (highest)—strongly suggests a synergistic effect where Ag-ZnO-coated curcumin significantly enhances the therapeutic action of the ointment.

The acceleration of wound closure was quantified and showed statistically significant differences, as detailed in Table 5. The healing time for soft wounds was reduced from 48.8 days with standard care to 12.3 days with the Ag-ZnO-curcumin treatment (*p* = 1.33 × 10^−7^). For medium wounds, the healing time dropped dramatically from 90.6 days to 19.6 days (*p* = 3.79 × 10^−5^). Preliminary data for the unloaded nanoparticle group (Group B) indicated a mean healing time intermediate between Groups A and C for both wound types, further supporting the role of curcumin as a critical active component. These *p*-values, being well below the significance threshold of 0.001, provide robust evidence that the observed acceleration is not due to chance.

The results of this randomized controlled trial confirm the high efficacy of Ag-ZnO nanoparticles modified with curcumin for promoting the healing of challenging wounds. The significant reduction in healing time and the higher rate of complete closure, especially in hard-to-heal wounds, underscore the therapeutic potential of this formulation. These findings align with previous clinical case reports [44,45,46] and suggest that curcumin-functionalized Ag-ZnO nanoparticle dressings are a promising and potent alternative to conventional silver-based dressings for managing both acute and chronic wounds.

**Figure 10:**
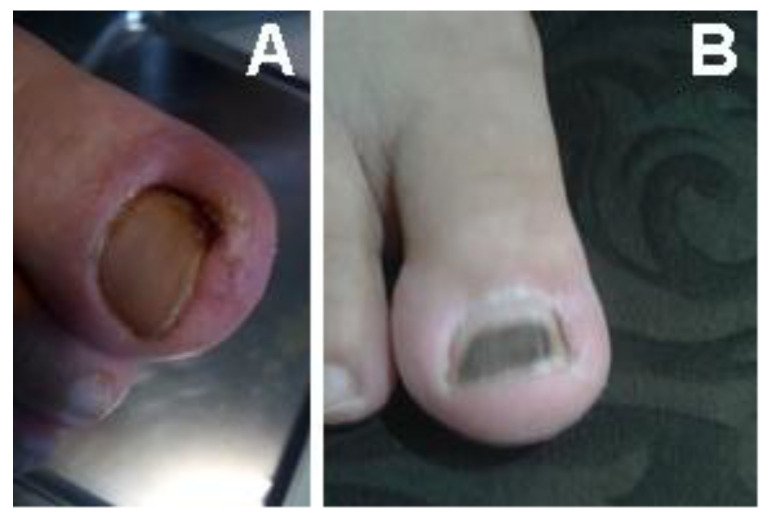
A patient with an infection on the big toe. (**A**) Before (**B**) after treatment within one week.

**Figure 11:**
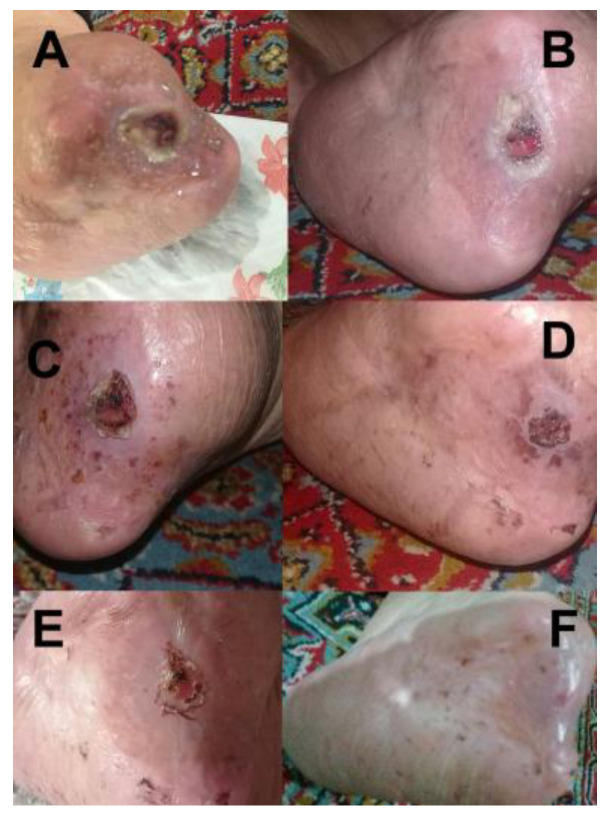
Wound-healing progression of a patient (**A**) before treatment, (**B**) 3 days, (**C**) 7 days, (**D**) 9 days, (**E**) 11 days, and (**F**) 14 days after treatment.

The patient presented in Figure 10 was referred to the Diabetes Association Clinic in Rasht for treatment of an infection on the toe. Following the administration of our medicine, a significant improvement was observed within one week, as demonstrated in Figure 10B, which showcases the toe in its healthy state at that time. The clinic received a patient who had a wound on the foot in Rasht. The patient was provided with the appropriate medicine for treating foot wounds (Figure 11) and underwent the necessary treatment. It is noteworthy that after two weeks, the wound exhibited significant progress and had completely healed. The wound-healing process corresponding to the wound is illustrated in Figure 11A–F.

**Figure 12:**
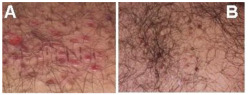
A patient with a wound on the back. (**A**) Before (**B**) After treatment.

The wound-healing process is The Tabriz Diabetes Clinic received a referral for a patient who had a wound on his back. The patient used the medicine that had been prepared promptly (Figure 12). It is noteworthy that the patient showed remarkable progress and fully recovered within just 4 days.

## 4. Conclusions

This study demonstrates the significant therapeutic potential of Ag-ZnO nanoparticles surface-functionalized with curcumin for promoting wound healing and combating bacterial infections. Through a strategy that combines computational design, robust in vitro and in vivo testing, and a controlled clinical trial, we established that curcumin-loaded nanoparticles exhibit potent, broad-spectrum antibacterial activity and dramatically accelerate wound repair. A key finding, elucidated through comparative groups including unloaded Ag-ZnO nanoparticles, is that the enhanced efficacy is specifically due to a synergistic effect between curcumin and the nanoparticle matrix, rather than either component alone. Clinical results confirmed statistically significant and superior healing outcomes compared to both standard care and the nanoparticle control. The formulation also demonstrated excellent biocompatibility in animal models. These comprehensive findings strongly suggest that curcumin-modified Ag-ZnO nanoparticles are a safe, effective, and translational option for advanced wound dressings, providing a practical and robust solution for managing chronic and infected wounds.

## Figures and Tables

**Table 1:** Main characteristics of the chemicals and reagents used in this study.

Compound/Material	Chemical Formula	CAS Number	Molecular Weight (g/mol)	Purity (%)	Supplier
Curcumin	C_21_H_20_O_6_	458-37-7	368.38	≥98	Sigma-Aldrich (Darmstadt, Germany)
Silver acetate	AgC_2_H_3_O_2_	563-63-3	166.91	≥99	Sigma-Aldrich (Darmstadt, Germany)
Polyvinylpyrrolidone (PVP, Mw ≈ 40,000)	(C_6_H_9_NO)_n_	9003-39-8	~40,000	≥99	Sigma-Aldrich (Darmstadt, Germany)
Sodium borohydride	NaBH_4_	16940-66-2	37.83	≥98	Sigma-Aldrich (Darmstadt, Germany)
Zinc acetate dihydrate	Zn(CH_3_COO)_2_·2H_2_O	5970-45-6	219.50	≥98	Sigma-Aldrich (Darmstadt, Germany)
Sodium hydroxide	NaOH	1310-73-2	40.00	≥98	Sigma-Aldrich (Darmstadt, Germany)
1-Hexadecanol	C_16_H_34_O	36653-82-4	242.44	≥98	Sigma-Aldrich (Darmstadt, Germany)
Linseed oil (solidified form)	C_18_ fatty acids mixture	8001-26-1	—	≥98	Venus Ethoxyethers Pvt (Goa, India)
Carboxymethyl cellulose sodium salt (CMC)	(C_6_H_7_O_2_(OH)_2_CH_2_COONa)_n_	9004-32-4	250,000–700,000	≥99	Sigma-Aldrich (Darmstadt, Germany)
Ethanol	C_2_H_6_O	64-17-5	46.07	≥99.9	Sigma-Aldrich (Darmstadt, Germany)

**Table 2:** The interaction parameters for docking compounds against the cell wall protein of *S. aureus* (Protein A).

	Molecule	RMSD	S (Energy Score) kcal/mol	Interacting Residues
1	Curcumin	0.952	−4.914	Lys F124
2	Curcumin-Ag	0.845	−8.424	Lys F124, Glu H148
3	Curcumin-ZnO	1.216	−4.871	Lys F124
4	1-(4-methyl phenyl)-3-(3-nitrophenyl)-2-propane-1-one	1.265	−2.945	Glu H146
5	1-(4-methyl phenyl)-3-(3-nitrophenyl)-2-propane-1-one-Ag	1.027	−5.203	Glu J 146, Asn F 162, Asn I 162
6	1-(4-methyl phenyl)-3-(3-nitrophenyl)-2-propane-1-one-ZnO	1.277	−3.749	Glu J 146, Asn I 162

**Table 3:** The effect of synthesized nanomaterials on the examined bacteria.

Sample	Bacteria	Bacteria Number in First Solution	60 Seconds	120 Seconds	Reference Number
15 μg/L (Ag)/100 μg/mL (ZnO), modified with 0.5 g/L curcumin	*E. coli*	8 × 10^9^	78	50	EN1276
	*Staphylococcus aureus*	6.2 × 10^9^	92	83	EN1276
	*Enterococcus hirae*	6.8 × 10^9^	80	65	EN1276
	*Pseudomonas aeruginosa*	4.9 × 10^9^	105	70	EN1276
	Methicillin-resistant *Staphylococcus aureus* (MRSA)	6.6 × 10^9^	144	68	EN1276

**Table 4:** Wound Closure Over Time in the Rabbit Model (% of Original Area Remaining).

Post-Operative Day	Group 1: Untreated Control	Group 2: Unloaded Ag-ZnO	Group 3: Ag-ZnO-Curcumin
Day 0	100.0 ± 0.0%	100.0 ± 0.0%	100.0 ± 0.0%
Day 3	95.2 ± 3.1%	91.5 ± 2.8%	82.4 ± 4.2%
Day 7	78.6 ± 5.5%	70.3 ± 4.9%	45.8 ± 6.1%
Day 10	55.1 ± 7.2%	45.2 ± 6.0%	15.3 ± 3.5%
Day 14	30.5 ± 8.4%	20.8 ± 5.7%	3.2 ± 1.8%
Time to Complete Closure (days)	21.4 ± 2.1	18.2 ± 1.8	12.5 ± 1.3

Data presented as mean ± standard deviation (n = 6 wounds per group). The Ag-ZnO-Curcumin group showed a statistically significant difference (*p* < 0.01) from both control groups starting from Day 3.

**Table 5:** Comparison of Mean Wound-Healing Times by Treatment Group.

Wound Type	Group A: Standard Care (Days)	Group B: Unloaded Ag-ZnO (Days)	Group C: Ag-ZnO-Curcumin (Days)	*p*-Value (A vs. C)
Soft	48.8 ± 5.2	38.5 ± 4.1	12.3 ± 2.1	1.33 × 10^−7^
Medium	90.6 ± 12.5	80.2 ± 6.8	19.6 ± 3.5	3.79 × 10^−5^

Data presented as mean ± standard deviation.

## Data Availability

We provide information on the search routines used to locate and then download those records. Those instructions allow an interested party with a suitable license to those databases to regenerate comparable datasets. For any required information, please contact rostami@tabrizu.ac.ir.
